# Fluorescence-Based Soil Survival Analysis of the Xenobiotic- and Metal-Detoxifying *Streptomyces* sp. MC1

**DOI:** 10.3390/ijms27010093

**Published:** 2025-12-21

**Authors:** Juan Daniel Aparicio, Victoria Guadalupe Gonzalez Holc, Cristhian Gabriel Pappalardo, Sylvie Lautru, Jean-Luc Pernodet, Marta Alejandra Polti

**Affiliations:** 1 Planta Piloto de Procesos Industriales Microbiológicos (PROIMI), Consejo Nacional de Investigaciones Científicas y Tecnológicas (CONICET), Av. Belgrano y Pasaje Caseros, Tucumán 4000, Argentina; jdaparicio@conicet.gov.ar (J.D.A.); guadalupegonzalezholc@gmail.com (V.G.G.H.); marta.polti@conicet.gov.ar (M.A.P.); 2 Facultad de Bioquímica, Química y Farmacia (FBQF), Universidad Nacional de Tucumán (UNT), Ayacucho 491, Tucumán 4000, Argentina; pcristhiangabriel@gmail.com; 3 Université Paris-Saclay, CEA, CNRS, Institute for Integrative Biology of the Cell (I2BC), 91198 Gif-sur-Yvette, France; sylvie.lautru@i2bc.paris-saclay.fr; 4 Facultad de Ciencias Naturales e Instituto Miguel Lillo, Universidad Nacional de Tucumán, Miguel Lillo 205, Tucumán 4000, Argentina

**Keywords:** conjugation, green fluorescent protein, lindane, chromium, bioremediation

## Abstract

*Streptomyces* sp. MC1, a bacterium isolated from an environment contaminated with organic and inorganic pollutants, can reduce chromium and degrade lindane, making it a promising candidate for bioremediation. However, a major challenge in bioremediation trials is monitoring bacteria survival in soil. To assess the survival of *Streptomyces* sp. MC1 during bioremediation, we introduced fluorescence tagging and a selectable marker into this strain by intergeneric conjugation from *Escherichia coli*. Conjugation assays were performed using two *E. coli* strains (ET12567/pUZ8002 or S17-1) and *Streptomyces* sp. MC1 (spores or mycelium). The integrative plasmid pSC001, carrying a gene encoding the monomeric green fluorescent protein (mGFP), was used. Various donor and recipient concentrations were tested and the presence of MgCl_2_ or CaCl_2_ during conjugation was also evaluated. Optimal conditions included low concentrations of both *Streptomyces* sp. MC1 spores and *E. coli* S17-1, with MgCl_2_ in the medium. Exconjugants were analyzed, confirming plasmid site-specific integration and mGFP expression. In bioremediation assays with soils co-contaminated with Cr(VI) and lindane, fluorescence-tagged *Streptomyces* sp. MC1 successfully demonstrated survival over 28 days. Our results, combined with the availability of the *Streptomyces* sp. MC1 genome sequence, will facilitate further characterization of this strain’s features and accelerate its development for bioremediation applications.

## 1. Introduction

*Streptomyces* sp. MC1, first isolated and characterized by Polti et al. [1], was recovered from sugarcane stems collected in an agricultural area adjacent to a drainage channel of a Cu-filter plant (Ranchillos, Tucumán—Argentina) and characterized by the presence of mixed contamination by both organic and inorganic pollutants. According to the comparative genomic analysis reported by Sineli et al. [2], *Streptomyces* sp. MC1 is phylogenetically distinct from typical soil-dwelling *Streptomyces* and clusters with strains exhibiting plant-associated traits (e.g., cellulose degradation), supporting its classification as an endophyte while not excluding its ability to survive and remain active in soil environments. In previous studies, *Streptomyces* sp. MC1 also demonstrated the ability to reduce high concentrations of Cr(VI) in contaminated soils under mesocosm conditions, both as a free-living bacterium and when associated with maize plants, confirming its functional activity outside plant tissues [3]. Culture experiments in liquid media and soil artificially co-polluted with Cr(VI) and γ-1,2,3,4,5,6-hexachlorocyclohexane (lindane) revealed the potential of this strain for bioremediation processes. Indeed, it exhibited remarkable resistance to high metal concentrations, the ability to reduce Cr(VI) to Cr(III), and the capacity to degrade lindane [4]. In a more recent study, *Streptomyces* sp. MC1 was combined with other actinomycetes to form a consortium that was successfully applied to the bioremediation of soil anthropogenically contaminated with Cr(VI) and lindane, highlighting the synergistic benefits of multi-strain approaches [5].

A critical challenge in bioaugmentation is the survival and activity of introduced strains in harsh environmental conditions. Traditional cultivation methods often lack the precision to distinguish them from native microbes, making it difficult to accurately track their performance [6]. Recent advances in molecular tools have revolutionized strain monitoring in environmental samples, enabling for more precise tracking of bioremediation inocula. For instance, DNA-based approaches, such as re-isolation of the strain followed by Random Amplified Polymorphic DNA Polymerase Chain Reaction (RAPD-PCR) analysis, have proven effective for strain tracking [7]. Although this technique has been widely and successfully applied, its complexity often limits its use in routine application. To address these limitations, biomarkers or marker genes, have emerged as powerful tools for tracking bacteria in natural environments, providing distinct traits for easy identification and monitoring. Among these methods, fluorescence labeling has become a promising alternative for more precise tracking [8]. For its successful implementation, however, such approaches require the ability to genetically modify bacterial strains to ensure the stable integration of fluorescent markers. This strategy holds significant potential to improve strain monitoring and enhance the evaluation of bioremediation efficiency in complex environmental settings.

The first important step in the genetic engineering of *Streptomyces* is the efficient introduction of heterologous DNA into host cells. Achieving this requires DNA delivery methods specifically tailored to each particular strain. Methods for foreign DNA delivery into *Streptomyces* include protoplast transformation, electroporation, transfection and intergeneric conjugation [9]. Among these methods, intergeneric conjugation is now the most commonly used and the one allowing introduction of DNA in the widest range of strains. Efficient DNA introduction protocols have been successfully developed for a few well-studied model strains, such as *S. coelicolor*, *S. lividans*, *S. griseus*, *S. avermitilis*, *S. venezuelae* and *S. albus* [10]. However, for less characterized and more recalcitrant strains, substantial adaptation and optimization is often required to ensure reliable DNA delivery and subsequent genetic manipulation [11]. For example, Mazodier et al. [12] were able to efficiently transform *S. lividans*, *S. coelicolor*, *S. pristinaespiralis*, and *S. viridochromogenes* with the conjugation method they developed, using *E. coli* S17-1 as donor strain. However, the method did not work on *S. parvulus* and *S. hygroscopicus*. Fouces et al. [13] and Qin et al. [14] optimized two different conjugation methods for *S. parvulus* and *S. hygroscopicus*, using the methylation-deficient *E. coli* ET12567/pUZ8002 as donor strain with the plasmid pUZ8002 providing mobilization functions.

The addition of MgCl_2_ in the conjugation medium is widely used [13], since Mg^2+^ promotes the conjugation process by positively charging the cell envelop and allowing easy incorporation of the (negatively charged) DNA. Although MgCl_2_ was initially used to improve conjugation efficiency, Yu and Tao [15] tested the effect of other salts: NaCl, Ca(NO_3_)_2_ and CaCl_2_, and found that if MgCl_2_ efficiently promoted DNA incorporation, CaCl_2_ did so as well, even to a greater extent with some strains. Thus, Wang and Jin [16] demonstrated that CaCl_2_ allowed obtaining a greater number of exconjugants with different streptomycetes. However, for many other *Streptomyces* strains, Mg^2+^ remains the most efficient cation [17,18].

Initially, interspecific conjugation was performed using *Streptomyces* spores, whose germination could be activated by thermal shock [10]. This procedure is the most efficient for the majority of *Streptomyces* strains. However, in some cases, the use of young mycelium proved to be more practical. For example, *S. rimosus* R7 and M4018 are heat-sensitive actinobacteria, so exposure to high temperatures reduces the ability of germinated spores to participate in conjugation [19]. Another example is *S. peucetius*, whose sporulation efficiency is low, so conjugation using spores is not a viable option [20]. An extreme case is that of *S. rimosus* R6-593, which, since it does not form spores, can only be transformed by conjugation using mycelium [19]. Beyond these exceptions, in some heat-resistant and spore-forming *Streptomyces* strains, the use of mycelium is simply more effective, as in *S. kanamyceticus* [18], while in others, as in *S. lividans*, it is more efficient to use spores [21].

The number of CFUs (colony forming units) of the donor and recipient and their ratio are other critical parameters in *Streptomyces* intergeneric conjugation [18]. For conjugation systems between *E. coli* ET12567/pUZ8002 and *S. rimosus*, *S. coelicolor*, *S. lavendulae* and *S. venezuelae*, the optimal number of CFU were 10^8^:10^6^, 10^7^:10^7^, 10^8^:10^8^ and 10^8^:10^8^, respectively [16,22]. However, for conjugation systems between *E. coli* S17-1 and *S. viridochromogenes*, *S. hygroscopicus*, *S. fradiae* or *S. aureofaciens*, the optimal conjugation ratios were 10^9^:10^9^, 10^8^:10^8^, 10^8^:10^9^ and 10^8^:10^8^, respectively [18].

In view of the above methodological peculiarities, it is clear that there is no universal protocol for intergeneric conjugation for the genus *Streptomyces*, so it is necessary to optimize the process conditions for each particular *Streptomyces* strain. In this context, this study aims to set up and optimize the intergeneric conjugation protocol for *Streptomyces* sp. MC1 using *Escherichia coli* as the donor strain. Furthermore, this work leverages fluorescence-based tools to address a long-standing challenge in monitoring bacterial survival and performance during bioremediation trials of soils. This dual approach not only refines genetic engineering strategies for *Streptomyces* but also enhances our ability to evaluate their environmental impact in real-world applications.

## 2. Results and Discussion

### 2.1. Choice of the Vector and Prediction of Its Integration Site in Streptomyces sp. MC1 Genome

**Figure 1:**
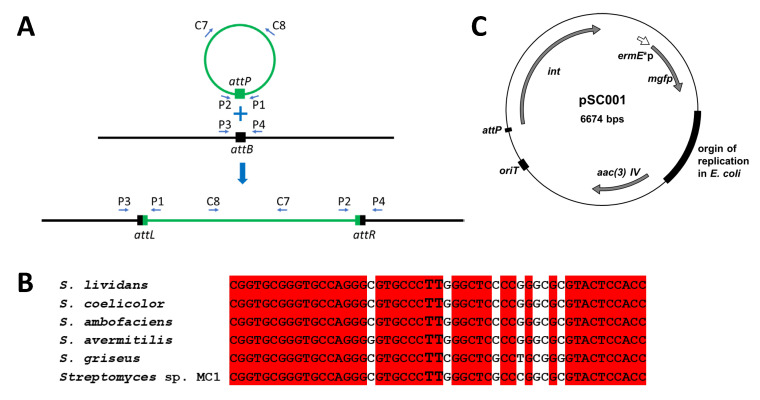
Site-specific integration of a *ϕC31* derived vector. (**A**): schematic representation of the site-specific integration of a *ϕC31* derived vector, the position of the primers C7, C8, P1, P2, P3, P4 are indicated by small arrows; (**B**): alignment of the predicted *ϕC31* attachment site *attB* in *Streptomyces* sp. MC1 with those of other *Streptomyces*, the two Ts constituting the core site in which recombination takes place are indicated by larger letters, the red background indicates conserved nucleotides; (**C**): schematic map of the vector pSC001.

As our final goal was the introduction and stable maintenance in *Streptomyces* sp. MC1 of a gene encoding a fluorescent protein together with an easily selectable antibiotic resistance marker, we selected a vector capable of efficient genomic integration. Over the past decades, several vectors have been developed based on the site-specific integration systems of temperate actinomycete bacteriophages [23]. These vectors integrate into the host genome via site-specific recombination between the phage attachment site *attP* and the bacteria attachment site *attB*, leading to the formation of the *attL* and *attR* sites (Figure 1A). The recombination event is promoted by an integrase. Among these integrative vectors, those derived from the phage *ϕC31* are widely used. In *Streptomyces*, the *ϕC31 attB* sites which have been characterized are located within a gene encoding a pirin-like protein which is present in most *Streptomyces* genomes (e.g., SCO3798 in *Streptomyces coelicolor* A3(2), protein accession N° CAC08479.1). Such a gene is indeed found in the genome of *Streptomyces* sp. MC1 (NCBI reference sequence NZ_JADWOR000000000.1 GI:194098455) and the encoded protein (Accession N° WP_196944705) presents 92% identity with the one from *S. coelicolor* A3(2). The *attB* site of *ϕC31* has been characterized in several *Streptomyces* strains [24]. A sequence highly similar to the ones of these *attB* sites is present within the pirin-like encoding gene from *Streptomyces* sp. MC1. In particular, the two Ts, where the recombination event is taking place, are conserved (Figure 1B). Therefore, the integration of vectors using the *ϕC31* site-specific recombination system should be possible at this predicted *attB* site in *Streptomyces* sp. MC1 genome. The plasmid pSC001 [25] (Figure 1C) was chosen as (i) it integrates in *Streptomyces* using the *ϕC31* site-specific recombination system, (ii) its presence can be selected with apramycin, an antibiotic to which *Streptomyces* MC1 is sensitive, (iii) it contains a gene encoding a fluorescent protein and (iv) it has previously been used to successfully track a *Streptomyces* strain in soil samples [25].

### 2.2. Transformation Efficiencies of E. coli ET12567/pUZ8002 and E. coli S17-1

The transformation efficiency achieved by electroporation differed significantly between the two strains. *E. coli* S17-1 showed a significantly higher transformation efficiency (9.88 × 10^7^ CFU mL^−1^) than *E. coli* ET12567/pUZ8002 (2.82 × 10^6^ CFU mL^−1^) (*p* < 0.05).

*E. coli* ET12567 harbors the plasmid pUZ8002 that provides the mobilization functions and carries the resistance markers *kan* and *chl*, conferring resistance to kanamycin and chloramphenicol, respectively [11]. In contrast, *E. coli* S17-1 contains the mobilization genes integrated within its chromosome [26]. This difference directly impacts bacterial growth conditions, as *E. coli* ET12567/pUZ8002 is cultivated in the presence of kanamycin and chloramphenicol to ensure the maintenance of pUZ8002, while *E. coli* S17-1 is cultivated without antibiotic selection.

The metabolic burden of antibiotic resistance can reduce bacterial growth rates and, consequently, post-transformation recovery efficiency [11], which may explain why lower efficiency rates were consistently observed with *E. coli* ET12567/pUZ8002. Nevertheless, both *E. coli* strains were tested during the optimization of the conjugation system.

### 2.3. Effect of Temperature on Sporulation, Pigmentation and Aerial Growth of Streptomyces sp. MC1 on Solid Media

Given the diversity of actinobacteria, a culture medium that supports robust spore production in one strain may be suboptimal for another [27]. Four media, commonly employed in the preservation and sporulation of *Streptomyces* were tested: International *Streptomyces* Project medium 4 (ISP4), Casein Starch Agar (CSA), Soy Flour Mannitol (SFM), and Medium for Production 5 (MP5).

The effect of temperature on spore production by *Streptomyces* sp. MC1 grown for 7 days in these different culture media was studied. The relationship between sporulation and pigmentation and aerial growth was also evaluated (Figure S1, Table 1).

At 50 °C, the rapid dehydration of the different solid culture media did not allow the growth of *Streptomyces* sp. MC1 (Figure S1).

Sporulation of *Streptomyces* sp. MC1 (Figure S1, Table 1a) was highest when cultured on CSA and incubated at 40 °C. Spore production was considerably lower on the other culture media. In ISP4 the highest spore production was achieved at 30 °C, whereas in SFM and MP5 sporulation was maximal at 35 °C.

On solid culture media, *Streptomyces* sp. MC1 displayed an inverse correlation between aerial growth (Table 1b) and sporulation (Table 1a); enhanced spore formation was correlated with a reduction in the remaining aerial mycelium. In ISP4, aerial growth of *Streptomyces* sp. MC1 was maximum at 40 and 45 °C. In CSA, aerial growth was maximum at 30 °C. In SFM and MP5, higher aerial growth was observed at 45 °C.

The pigmentation produced by *Streptomyces* sp. MC1 (Table 1c) on the different culture media correlated with the level of sporulation observed (Table 1a), as conditions that promoted higher pigment production, also increased spore production. Specifically, in ISP4, the highest pigmentation was obtained when the cultures were incubated at 30 °C, whereas in CSA, the highest pigment production was observed at 45 °C. In MP5 and SFM, pigmentation was higher at 35 °C.

Based on these results, the solid culture medium CSA and the incubation temperature 40 °C were selected as optimal conditions for spore production to be used in subsequent studies.

The distinction between substrate mycelium and aerial mycelium of *Streptomyces* goes beyond their location, they have long been recognized as physiologically different [28]. The aerial mycelium is thicker and less branched than the substrate mycelium, and while division and differentiation of the former lead to spore formation, the latter is primarily responsible for pigmentation. Moreover, the *Streptomyces* life cycle has proven to be more complex than previously thought, as growth, sporulation, and pigmentation are closely interrelated. In particular, it has been shown that higher sporulation correlates with increased pigment production, whereas enhanced aerial mycelium formation is often accompanied by a loss of pigmentation [29]. This relationship was further confirmed by the spore production assays conducted in this study.

### 2.4. Optimization of Intergeneric Conjugation Between E. coli and Streptomyces sp. MC1

For the optimization of intergeneric conjugation conditions between *E. coli* donor strains (ET12567/pUZ8002 or S17-1) and *Streptomyces* sp. MC1 (spores or mycelium), the influence of donor/recipient ratios, and of the presence of divalent cations (MgCl_2_ and CaCl_2_) on conjugation efficiency was systematically evaluated.

Initially, the intergeneric conjugation was attempted using the traditionally employed medium, SFM [30]. However, no apramycin resistant exconjugants were obtained under any of the conditions tested. Therefore, the previously selected medium, CSA, was used instead. With this medium, apramycin resistant clones of *Streptomyces* sp. MC1 were obtained after conjugation in all conditions tested, except in the controls experiments in which the *E. coli* strains lacking the shuttle plasmid pSC001 were used. Further characterization of the apramycin resistant exconjugants (see below 2.5) confirmed that they contained pSC001. Therefore, the frequency of plasmid transfer was calculated as the number of apramycin resistant exconjugants divided by the initial number of recipient CFUs.

The results (Table 2 and Table 3) showed that conjugation frequencies using *Streptomyces* sp. MC1 spores were higher when the lowest concentrations of both donor and recipient strains were used. In contrast, when mycelium was used as the recipient, a higher concentration of donor strain cells was required to achieve optimal conjugation efficiency.

In conjugation media supplemented with 10 mM MgCl_2_, the conjugation efficiency between *E. coli* ET12567/pUZ8002 and *Streptomyces* sp. MC1 was higher when spores of the recipient strain were used. In contrast, no significant differences in conjugation efficiency were observed between *E. coli* S17-1 and *Streptomyces* sp. MC1, regardless of whether spores or mycelium were used as the recipient. The use of CaCl_2_ 60 mM in the conjugation medium favored plasmid transfer between both donor strains and mycelium of *Streptomyces* sp. MC1, resulting in the highest transformation efficiencies. However, this efficiency was significantly reduced when spores of the recipient strain were used, reaching the lowest transformation values.

Moreover, plasmid transfer efficiency was consistently higher when *E. coli* S17-1 was used as the donor strain (compare the values in Table 2 and Table 3).

In addition, it is important to highlight a distinctive morphological characteristic of the exconjugants obtained. Colonies of the wild-type *Streptomyces* sp. MC1 strain are flat, extremely hard and initially exhibit a smooth and opaque surface (young, unsporulated cultures), and later, the surface becomes rough, developing a “snowy” appearance (sporulated cultures). Its color transitions from white, gray and yellow, depending on the age and conditions of the culture. However, when grown on CSA conjugation medium containing MgCl_2_ or CaCl_2_, nalidixic acid and apramycin, the colony morphology changed significantly. The exconjugant colonies were yellow, raised, creamy in consistency and their surface was smooth and shiny. In addition, no spores were observed throughout the incubation period, suggesting that sporulation was inhibited under this culture conditions. Complementary tests were performed to identify which of the additives were responsible for the observed morphological changes. These revealed that MgCl_2_ and CaCl_2_ were the factors responsible for the alterations. Moreover, after transferring the exconjungants to CSA medium without additives, they recovered their original morphology, confirming that the changes were temporary and induced by the culture conditions.

While in some species the donor/recipient ratio does not affect conjugative efficiency, as are the case of *Streptomyces coelicolor* [11] and *Streptomyces nodosus* [31], in other cases, a certain ratio of donor cells to number of recipient cells is required to achieve the highest frequency, as is the case of *Streptomyces lincolnensis* [32], *Streptomyces diastatochromogenes* 1628 [33], *Streptomyces natalensis* [34] and *Streptomyces noursei* [35]. For *Streptomyces* sp. MC1, our results show that the donor/recipient ratio affects transformation efficiency.

In many *Streptomyces* strains possessing a methylation-specific restriction system, using a donor strain that methylates DNA has the effect of drastically reducing the number of exconjugants obtained by intergeneric conjugation [11]. Our results shown that *Streptomyces* sp. MC1 lacks a methylation-specific restriction system, as conjugation was more efficient with *E. coli* S17-1 (methylation competent) than with *E. coli* ET12567 (methylation deficient) as donor. This finding makes *E. coli* S17-1 the ideal donor for intergeneric conjugation with *Streptomyces* sp. MC1.

Although mycelium-based conjugation yielded higher initial efficiencies, it posed significant methodological challenges [21]. First, obtaining the mycelium suspension requires disaggregation using a manual Potter-Elvehjem homogenizer, which might introduce contamination. Second, the quantification of the suspension depends on the degree of disaggregation (the more disaggregated, the greater the resulting CFU). Finally, after conjugation, the rapid growth of *Streptomyces* sp. MC1 as mycelium is accompanied by strong yellow pigmentation, complicating the counting and picking of exconjugants to start new cultures.

Based on the above, it was decided to select the most efficient condition obtained with spores, i.e., the intergeneric conjugation between *E. coli* S17-1 (10^7^ CFU) and *Streptomyces* sp. MC1 spores (10^7^ CFU) in medium containing 10 mM MgCl_2_. Transformation efficiencies achieved were consistent with, or comparable to, those previously reported for other *Streptomyces* species, highlighting the robustness of the applied methodology [16,22].

### 2.5. Characterization of the Exconjugants

Three tests were performed to characterize the *Streptomyces* sp. MC1 exconjugants obtained.

**Figure 2:**
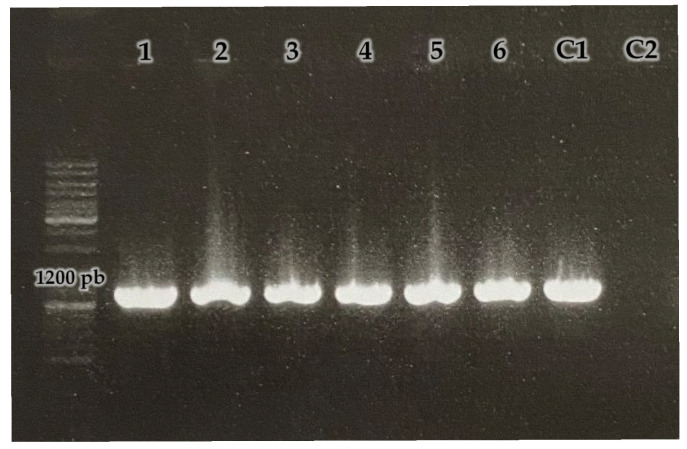
0.8% agarose gel electrophoresis of PCR amplification products of the *mgfp* gene using primers C7 and C8. 1–6: exconjugants of *Streptomyces* sp. MC1, (C1) pSC001, (C2) wild-type *Streptomyces* sp. MC1.

The first test assessed the presence of the pSC001 plasmid in the exconjugants. Total DNA was extracted from six exconjugants. Primers C7 and C8 were used to amplify the *mgfp* gene (Figure 2). Plasmid pSC001 was used as a positive control, and DNA from the wild-type *Streptomyces* sp. MC1 strain was used as a negative control. An amplification product of the expected size (1200 bp) was obtained when DNA from all six exconjugants and the positive control were used.

**Figure 3:**
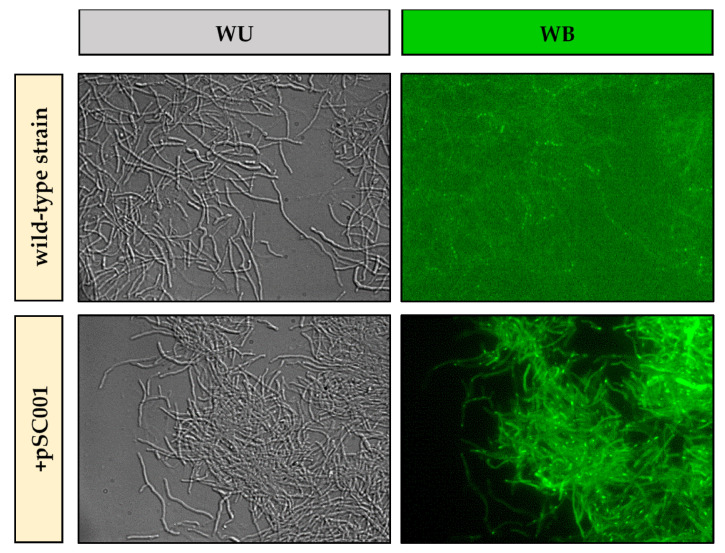
Visualization of *Streptomyces* sp. MC1 (wild-type strain) and an exconjugant (+pSC001) at 400×. WU: without filter; WB: blue filter.

The presence of green fluorescence in the hyphae of the exconjugants would indicate that, in addition to a successful plasmid transfer, the *mgfp* gene was expressed by the recipient strain. The second test consisted of evaluating mGFP expression using fluorescence microscopy (Figure 3). Visualization of the exconjugants was initially performed without a light filter (WU) to establish the reference image. Subsequently, a WB filter (green fluorescence excited by blue light) was used, revealing that, unlike the wild-type strain, the exconjugants emitted uniform green fluorescence along their hyphae. This confirmed that not only the pSC001 plasmid was successfully transferred, but mGFP was also expressed by *Streptomyces* sp. MC1. Intense green dots observed in both strains likely result from natural autofluorescence concentrated at hyphal tips, branches, or overlapping hyphae [36].

**Figure 4:**
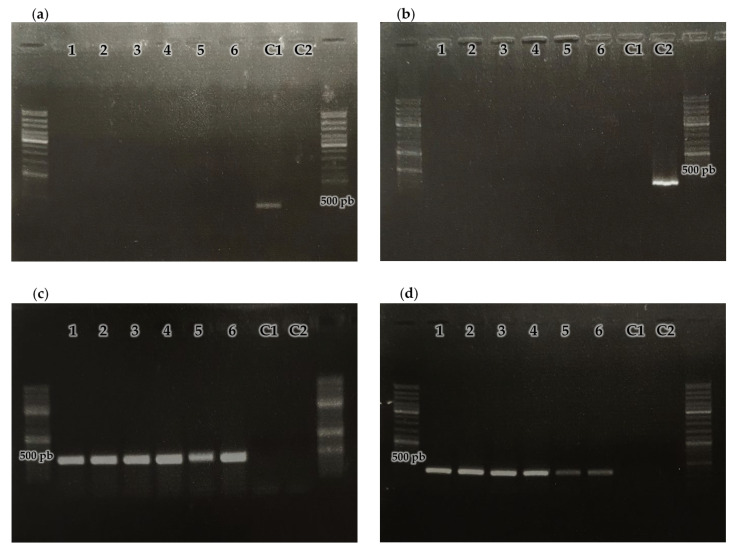
0.8% agarose gel electrophoresis of PCR amplification products of the sites: (**a**) *attP* using primers P1 and P2, (**b**) *attB* using primers P3 and P4, (**c**) *attL* using primers P1 and P3, and (**d**) *attR* using primers P4 and P2. 1–6: *Streptomyces* sp. MC1 exconjugants, (C1) pSC001, (C2) wild-type *Streptomyces* sp. MC1 strain.

To demonstrate the site-specific integration of the plasmid into the bacterial chromosome, a third test was carried out. The pSC001 plasmid contains the *ϕC31* integration system, which allows site-specific integration into the host chromosome (Figure 1). To verify that the *Streptomyces* sp. MC1 exconjugants had integrated the plasmid into their chromosome at the predicted site, four PCR were performed (Figure 4). DNA extracted from the six exconjugants and two controls (plasmid pSC001 and wild-type strain DNA) were used as templates. For the amplification of the *attB* region, an amplification product was obtained only when DNA from the wild-type strain was used as a template. This indicates that all the chromosomal *attB* sites might be occupied by pSC001 in the exconjugants. For the amplification of the *attP* region, an amplicon was observed only when the plasmid pSC001 was used as a template. This indicates that in the exconjugants site-specific recombination between *attP* and the predicted *attB* has occurred, leading to the formation of the *attL* and *attR* sites (Figure 1). This was confirmed because *attL* and *attR* amplification products were obtained only from the exconjugants DNA. All these results demonstrate that the plasmid pSC001 was site-specifically integrated into the chromosome of all the exconjugants studied and that the genes it carries are expressed.

### 2.6. Successful Monitoring of Streptomyces sp. MC1 in a Bioremediation Assay

A previous mesocosm study demonstrated the ability of MC1 to remediate Cr(VI) and lindane in soil and soil–plant systems [3]. Here, we reassessed its survival to confirm that these functions remain stable after fluorescent tagging, ensuring that the genetic modification does not compromise its environmental performance.

We now have a *Streptomyces* sp. MC1 strain harboring pSC001, making it resistant to apramycin and fluorescently labeled. This strain was used to inoculate soil samples contaminated with lindane and Cr(VI).

**Figure 5:**
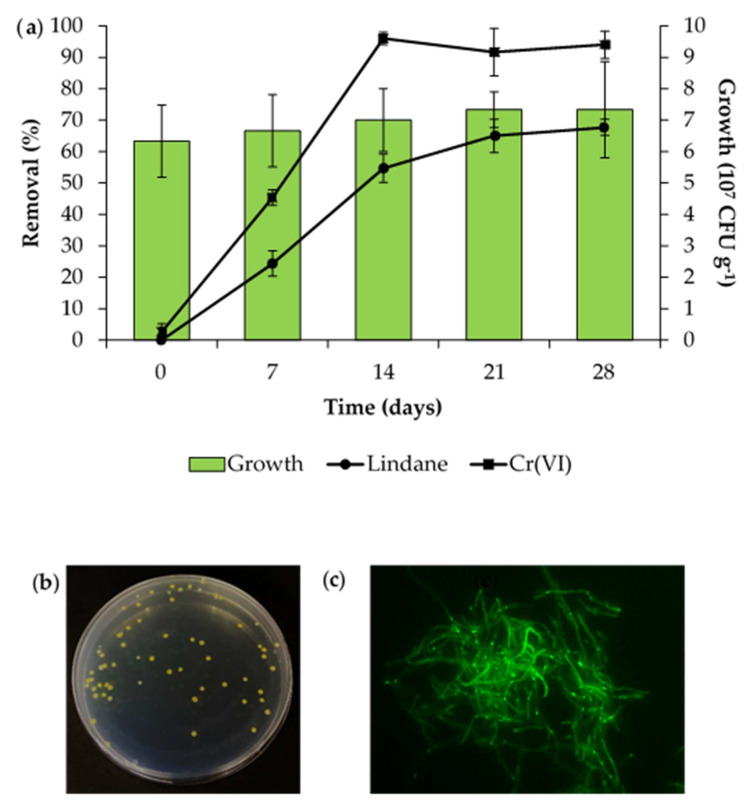
Bioremediation of anthropologically co-contaminated soil by *Streptomyces* sp. MC1 + pSC001. (**a**) Lindane and Cr(VI) removals and strain growth; (**b**) Re-isolation of *Streptomyces* sp. MC1 from soil mesocosms; and (**c**) fluorescence of a re-isolated colony at 400×.

Lindane and chromium removal profiles were then evaluated (Figure 5a). The initial concentrations of lindane and Cr(VI) were 556 µg kg^−1^ and 197 mg kg^−1^, respectively. Lindane removal increased up to a maximum of 65% in day 21, and then remained constant. On the other hand, maximum Cr(VI) removal was reached on day 14, being more than 96%. No removal of either compound was observed in the non-inoculated controls.

In a previous study, *Streptomyces* sp. MC1 exhibited similar pollutant-removal levels, although experimental conditions differed from those used here [37]. Thus, while our results indicate that pSC001 did not noticeably affect MC1 performance under the conditions tested, a definitive assessment would require experiments conducted under identical conditions.

To evaluate the survival of the strain, re-isolation was performed on CSA supplemented with nalidixic acid 50 µg mL^−1^, cycloheximide 50 μg mL^−1^, minocycline 15 µg mL^−1^, and Apr 50 μg mL^−1^ (Figure 5b). All the resistant colonies recovered exhibited green fluorescence, indicating that they contained pSC001. The successful expression of the *mgfp* gene confirms the stability of the features conferred by pSC001 (Figure 5c). This approach allowed the successful monitoring of the bioremediation agent in anthropologically co-contaminated soils.

This advancement represents a significant step forward, as it allows for precise, real-time tracking of *Streptomyces* sp. MC1 during bioremediation experiments in soils contaminated with Cr(VI) and lindane. In comparison, traditional approaches like RAPD-PCR [7], are more laborious, whereas fluorescence labeling offers a simplified, scalable, and highly reliable method for monitoring bacterial survival, distribution, and activity in complex environments. Moreover, this approach opens new possibilities to study the molecular mechanisms underlying the bioremediation process, many of which remain poorly understood.

## 3. Materials and Methods

### 3.1. Microorganisms and Plasmid

Two strains were used as donors: *E. coli* ET12567/pUZ8002 and *E. coli* S17-1:

- *E. coli* ET12567/pUZ8002 is a methylation-defective strain (*dam-* and *dcm-*) and lacks *E. coli*’s exogenous DNA restriction system (*hsdM* and *hsdR*). This allows efficient uptake of foreign DNA. In turn, homologous recombination is suppressed (*recF143*). This strain harbors the plasmid pUZ8002 that provides it with mobilization functions (*tra* genes) [11].
- *E. coli* S17-1 contains a derivative of the IncPα RP4 plasmid integrated into its chromosome, thus carrying the mobilization functions. This strain is also devoid of *E. coli* own exogenous DNA restriction system (*hsdR*) that allows efficient uptake of foreign DNA. In addition, homologous recombination is suppressed (*recA2*) [26].

In order to study its ability to be genetically transformed by intergeneric conjugation, *Streptomyces* sp. MC1, which was previously isolated and characterized in the Actinobacteria Biotechnology Laboratory of PROIMI [1], was used as a recipient strain.

The *E. coli/Streptomyces* shuttle plasmid pSC001 (Figure 1C) was used [25].

### 3.2. Streptomyces sp. MC1 Preparation

#### 3.2.1. Spore Production: Selection of Culture Medium and Optimum Temperature

Spore production of *Streptomyces* sp. MC1 was evaluated in different solid culture media, which were formulated for the growth and sporulation of actinomycetes:

- ISP4 [composition in g L^−1^: starch, 10; K_2_HPO_4_, 1; MgSO_4_·7H_2_O, 1; NaCl, 1; (NH_4_)_2_SO_4_, 2; CaCO_3_, 2; FeSO_4_·7H_2_O, 0.001; MnCl_2_·4H_2_O, 0.001; ZnSO_4_·7H_2_O, 0.001; agar; pH 7.0 ± 0.2)] (Sigma-Aldrich, Darmstadt, Germany) [38].
- CSA [composition in g L^−1^: starch, 10; casein, 1; K_2_HPO_4_, 0.5; agar, 15; pH 7.0 ± 0.2] (Sigma-Aldrich, Darmstadt, Germany) [4].
- SFM [composition in g L^−1^: mannitol, 20; soy flour, 20; agar, 15; pH 7.0 ± 0.2] (Sigma-Aldrich, Darmstadt, Germany) [30].
- MP5 [composition in g L^−1^: yeast extract, 7; NaCl, 5; NaNO_3_, 1; Glycerol, 45; MOPS, 20; agar, 15; pH 7.0 ± 0.2] (Sigma-Aldrich, Darmstadt, Germany) [39].

Different incubation temperatures (30, 35, 40, 45 and 50 °C) were tested.

A semi-quantitative characterization of sporulation, aerial growth and pigmentation was performed by stereo microscopic observation to select the appropriate culture medium and the optimal sporulation temperature.

*Streptomyces* sp. MC1 was seeded in Petri dishes containing the selected culture medium and incubated for 7 days at the previously determined optimal temperature. After incubation, 10 mL of sterile distilled water was added to each dish. Spores were gently dislodged from the surface with a sterile loop and collected using a sterile pipette. The suspension was filtered, the filtrate was placed in a centrifuge tube and shaken vigorously to break the spore chains. Subsequently, it was centrifuged at 8000× *g* for 10 min at 4 °C and the supernatant was separated immediately, in order to remove all solubilized compounds from the culture medium, which could reduce the longevity of the spores or inhibit their germination. The collected spores were resuspended in 1 mL of 20% glycerol and stored at −80 °C.

#### 3.2.2. Biomass Production

*Streptomyces* sp. MC1 mycelium was obtained after cultivation in 30 mL of Tryptic Soy Broth medium (TSB) [composition in g L^−1^: tryptone, 17; soy peptone, 3; NaCl, 5; K_2_HPO_4_, 2.5; glucose, 2.3; pH 7.0 ± 0.2] (Laboratorios Britania S.A., Buenos Aires, Argentina) where 10 µL of the spore suspension was inoculated (10^8^ spores). The culture was incubated in an orbital shaker (Innova®, New Brunswick Scientific, Edison, NJ, USA) at 200 rpm at 30 °C. After 24 h, the pellet was recovered by centrifugation (Eppendorf 5804R, Eppendorf, Hamburg, Germany) at 8000× *g* and washed twice with sterile distilled water. The mycelium suspension was disintegrated using a Potter-Elvehjem manual homogenizer (Fisher Scientific, Villebon-sur-Yvette, France). The biomass obtained was resuspended in sterile distilled water at a final concentration of 100 g L^−1^ and stored at 4 °C [4].

### 3.3. E. coli Transformation by Electroporation

For each donor *E. coli* strain (*E. coli* ET12567/pUZ8002 or *E. coli* S17-1), two tubes with electrocompetent cells were thawed on ice [40]. One of them was used to perform the transformation itself, for which 1 µL of plasmid pSC001 (1 ng) was placed. The other was used as a control, added with 1 µL of PCR-quality H_2_O.

The electroporation was performed using the Eppendorf Electroporator 2510 (Eppendorf SE, Hamburg, Germany) at 2500 V, 10 μFD, 600 Ω and a pulse time of ±6 ms. Then, the contents of each microtube were transferred to 15 mL capacity tubes, with 1 mL of LB medium [composition in g L^−1^: tryptone, 10; yeast extract, 5; NaCl, 10; pH 7.0 ± 0.2] pre-warmed to 37 °C, and incubated at 200 rpm at 37 °C for 2 h. Finally, the content of each tube was seeded by successive dilutions (10^−1^ to 10^−10^) on plates with solid LB medium containing Apr 50 µg mL^−1^. In the case of *E. coli* ET12567/pUZ8002, Km 25 µg mL^−1^ and Cm 25 µg mL^−1^ were also added. For the plasmid-free controls, an additional plate was made without Apr. After 24 h incubation at 37 °C, CFU mL^−1^ was determined and transformation efficiency was calculated as CFU obtained per ng of plasmid.

The transformation efficiencies with each method were statistically compared. For each *E. coli* strain, the protocol that yielded the highest transformation efficiency was determined.

### 3.4. Optimization of the Intergeneric Conjugation Between E. coli and Streptomyces sp. MC1

Intergeneric conjugation between *E. coli* (*E. coli* ET12567/pUZ8002 or *E. coli* S17-1) and *Streptomyces* sp. MC1 (spore or biomass) was performed as described by Zhang et al. [18] with some modifications. Different ratios of donor cell/recipient cell were evaluated by combining three cell quantities of the donor strain and three of the recipient strain.

On the one hand, *E. coli* pre-cultures were prepared in 1 mL of LB medium with the appropriate antibiotics for both *E. coli* strains containing the plasmid and for non-transformed strains, which were used as controls. Subsequently, each strain was cultured in 50 mL of LB (inoculated with 0.5 mL of the preculture) with the appropriate antibiotics and grown to an OD600 of 0.6. To remove the antibiotics, the cells were centrifuged (4000× *g*, 4 °C, 5 min) and washed twice with LB. They were then re-suspended in 5 mL of LB to obtain a concentration of 10^9^ CFU mL^−1^). Two dilutions were also prepared: 10^8^ and 10^7^ CFU mL^−1^.

On the other hand, 1 mL of *Streptomyces* sp. MC1 spore suspension was taken, washed twice and re-suspended in 1 mL of TSB medium. Subsequently, the suspension was heated at 50 °C for 10 min (to activate its germination), and then incubated at 37 °C for 2 h. When mycelium was used, no heating or incubation was performed prior to conjugation.

The conjugation was performed by mixing 100 μL *E. coli* cells (10^9^, 10^8^, or 10^7^ CFU mL^−1^) and spores or mycelium of *Streptomyces* sp. MC1 in the following amounts: 10^9^, 10^8^, or 10^7^ CFU. They were centrifuged (8500× *g*, 1 min) and most of the supernatant was removed to finally seed the pellet on CSA plates to which were added 10 mM MgCl_2_ or 60 mM CaCl_2_, since the effect of these salts on conjugation was also evaluated. Controls were made by mixing each *E. coli* strain without plasmid (10^8^ CFU) with spore and mycelium suspension (10^8^ CFU). Plates were incubated for 14–20 h at 30 °C and then covered with 3 mL of the SCN medium [composition in g L^−1^: starch, 10; casein, 0.3; KNO_3_, 2; MgSO_4_·7H_2_O, 0.05; K_2_HPO_4_, 2; NaCl, 2; CaCO_3_, 0.02; FeSO_4_·7H_2_O, 0.01; agar, 18; pH 7.0 ± 0.2] supplemented with nalidixic acid and apramycin in order to have a final concentration in the plate of 50 μg mL^−1^ for each antibiotic. They were incubated at 30 °C until the appearance of exconjugants (7–10 days). The frequency of plasmid transfer was calculated as the number of exconjugants divided by the number of recipient CFU.

Exconjugants were seeded on plates of CSA containing Apr 50 μg mL^−1^ and incubated for 7–10 d at 30 °C.

### 3.5. Characterization of the Exconjugants

For confirmatory tests, 6 exconjugants were selected and cultured in 1 mL TSB containing Apr 50 μg mL^−1^ at 30 °C for 24 h.

#### 3.5.1. Plasmid Incorporation Test by PCR

The mycelium of *Streptomyces* MC1 exconjugants was recovered by centrifugation (8500× *g*, 1 min) and resuspended in lysis buffer. DNA extraction was performed using the commercial Wizard^®^ Genomic DNA Purification Kit (Promega, Madison, WI, USA) after 2 h incubation at 37 °C.

The presence of the plasmid in *Streptomyces* MC1 exconjugants was verified by PCR, using the primers C7 (5′-CGGGCCTCTTCGCTATTAC-3′) and C8 (5′-TTATGCTTCCGGCTCGTATG-3′), which allow the amplification of a region of about 1200 bp encompassing the *mgfp* gene. The amplification conditions were as follows: 95 °C, 3 min/95 °C, 30 s; 58 °C, 30 s; 72 °C 1.5 min (30 cycles)/72 °C, 10 m. Plasmids were used as positive control and untransformed *Streptomyces* sp. MC1 DNA as negative control.

#### 3.5.2. Fluorescence

For visualization of the fluorescence emitted by the exconjugants, the obtained pellets were sampled, disaggregated with the aid of a needle and observed with an Olympus System BX60 microscope (Olympus Corporation, Tokyo, Japan) equipped with the appropriate filter sets. Images were recorded with an Olympus F-view II camera (Olympus Corporation, Tokyo, Japan) [41].

#### 3.5.3. Plasmid Integration Test by PCR

The integration of the plasmid into the *Streptomyces* MC1 chromosome through the *ϕC31* integration system was confirmed by performing four PCRs. Four primers were designed: P1 (5′-CTGCAGGCATGCAAGCTCTAGCGAT-3′), P2 (5′-CGCTTCGCTGAAATGCCCGACGAA-3), P3 (5′-CGGTTTCGAGGGCGAGGGCTTCC-3′), and P4 (5′-GGGAGGTTCACCCACAGCTGCA-3). The P1–P2 pair allowed amplification of the attachment site in the plasmid pSC001 (*attP* sequence), the P3–P4 pair allowed amplification of the attachment site in *Streptomyces* MC1 genome (*attB* sequence), and the P1–P3 and P2–P4 pairs allowed amplification of the *attL* and *attR* sequences (attachment sites in the chromosome after site-specific integration of pSC001), respectively, in the exconjugants. The amplification conditions were as follows: 95 °C, 3 min/95 °C, 30 s; 60 °C, 30 s; 72 °C 30 s (30 cycles)/72 °C, 10 min.

The size of the amplification products obtained was evaluated by electrophoresis in 2% agarose gel, stained with red gel. The molecular weight marker 1 kb DNA Ladder (Promega) was used.

### 3.6. Soil Bioremediation by Streptomyces sp. MC1 and Survival Test

Soil samples previously contaminated by anthropogenic activity with lindane and Cr(VI) were collected from Chicoana (25°06′19.3″ S, 65°31′09.7″ W) in the province of Salta, Argentina. The samples were subsequently conditioned following the methodology described by Aparicio et al. [5]. Glass pots were filled with 200 g of soil and humidity was fixed at 20% using sterile water. After that, the soils were inoculated with 2 g kg^−1^ of *Streptomyces* sp. MC1 biomass and mixed thoroughly to ensure a uniform distribution of the actinobacteria. Also, non-inoculated soils were used as controls. The soil pots were incubated for 28 days at 30 °C. Soils were aerated by mixing and moisture was monitored by weight difference once a week. A sample was taken at the beginning of the assay and every 7 days to determine lindane concentrations by a Gas Chromatography [5], to quantify Cr(VI) concentrations by atomic absorption spectrometry [5], and to perform the survival test.

Strain survival evaluation was carried out following the procedure described by Aparicio et al. [5]. Re-isolation from soil was performed on CSA supplemented with nalidixic acid (50 µg mL^−1^), cycloheximide (50 μg mL^−1^), and minocycline (15 µg mL^−1^). Apramycin (50 μg mL^−1^) was also added, as the exconjugants are resistant to this antibiotic. Microbial growth of the resistant colonies obtained was determined as CFU g^−1^, and their fluorescence evaluated under microscope as described in Section 3.5.2.

### 3.7. Statistical Analysis

The statistical analyses were conducted using R software (version 4.4.1), within the RStudio environment (version 2024.09.0+375). For all inferential tests, a *p*-value of less than 0.05 was considered statistically significant. Normality and homoscedasticity were assumed for the data. A two-way ANOVA was performed, followed by post hoc comparisons using the Tukey method where appropriate.

## 4. Conclusions

This study represents the first successful genetic engineering of *Streptomyces* sp. MC1, marking a significant step forward in the development of this strain for biotechnological applications. In the present work, we demonstrated that sporulation of *Streptomyces* sp. MC1 is optimized in CSA medium at 40 °C. Moreover, the best conditions for the conjugation between *E. coli* and *Streptomyces* sp. MC1 were established: lower concentration of spores of *Streptomyces* sp. MC1, higher concentration of *E. coli* and presence of MgCl_2_ in the CSA conjugation medium. Furthermore, the plasmid transfer efficiency was higher when the donor strain was *E. coli* S17-1 compared to *E. coli* ET12567/pUZ8002, demonstrating that *Streptomyces* sp. MC1 does not have a specific methylation restriction system. Additionally, *Streptomyces* sp. MC1 fluorescently labeled was successfully monitored in a bioremediation assay of soils anthropogenically contaminated with Cr(VI) and lindane, providing a novel tool for tracking bacterial dynamics in bioremediation. This study establishes, for the first time, an efficient system for intergeneric conjugation between *E. coli* and *Streptomyces* sp. MC1, enabling transformation and genetic manipulation of this strain, with great biotechnological potential. The remarkable tolerance of *Streptomyces* sp. MC1 to chromium, combined with the successful integration of the green fluorescent protein, provides the basis for its development as a biosensor, harnessing fluorescence as a real-time indicator of environmental chromium levels. These findings enhance the molecular toolset of *Streptomyces* sp. MC1, providing a robust platform to further explore its genetic and metabolic potential, while advancing its application in innovative strategies for environmental monitoring, bioremediation and other biotechnological processes.

## Figures and Tables

**Table 1:** (**a**) Sporulation, (**b**) Aerial growth, and (**c**) Pigmentation of *Streptomyces* sp. MC1 on different solid culture media: ISP4 (International *Streptomyces* Project medium 4), CSA (Casein Starch Agar), SFM (Soy Flour Mannitol), and MP5 (Medium of Production 5). Relative scale: + = low, ++ = moderate, +++ = high, ++++ = very high, and - = none.

(a) Sporulation					
Media	Temperature				
	30 °C	35 °C	40 °C	45 °C	50 °C
ISP4	++	+	-	-	-
CSA	++	+++	++++	+++	-
SFM	+	++	+	-	-
MP5	+	++	+	-	-
(b) Aerial growth					
Media	Temperature				
	30 °C	35 °C	40 °C	45 °C	50 °C
ISP4	+	++	+++	+++	-
CSA	+++	++	+	++	-
SFM	++	+	++	+++	-
MP5	++	+	++	+++	-
(c) Pigmentation					
Media	Temperature				
	30 °C	35 °C	40 °C	45 °C	50 °C
ISP4	++++	+++	++	+	-
CSA	++	+++	++++	++	-
SFM	+++	++++	++	+	-
MP5	++	++++	+++	+	-

**Table 2:** Intergeneric conjugation frequencies between *E. coli* ET12567/pUZ8002 and *Streptomyces* sp. MC1 in different conditions and with various donor/recipient ratios (CFU/plate) The mean values are given. Different letters indicate significant differences between means (*p* < 0.05).

MgCl_2_ 10 mM									
(a)	CFU/plate	*E. coli* ET12567/pUZ8002			(b)	CFU/plate	*E. coli* ET12567/pUZ8002		
		10^7^	10^8^	10^9^			10^7^	10^8^	10^9^
MC1 (spores)	10^7^	1.9 × 10^−6^ *d*	1.5 × 10^−6^ *e*	3.7 × 10^−7^ *jk*	MC1 (mycelium)	10^7^	1.0 × 10^−6^ *fg*	1.1 × 10^−6^ *f*	1.6 × 10^−6^ *e*
	10^8^	6.0 × 10^−7^ *i*	6.9 × 10^−7^ *hi*	5.3 × 10^−7^ *ij*		10^8^	7.7 × 10^−8^ *mnop*	1.1 × 10^−7^ *lmnop*	1.3 × 10^−7^ *lmnop*
	10^9^	8.8 × 10^−8^ *mnop*	7.4 × 10^−8^ *mnop*	6.6 × 10^−8^ *mnop*		10^9^	6.0 × 10^−9^ *p*	8.3 × 10^−9^ *op*	1.2 × 10^−8^ *op*
CaCl_2_ 60 mM									
(c)	CFU/plate	*E. coli* ET12567/pUZ8002			(d)	CFU/plate	*E. coli* ET12567/pUZ8002		
		10^7^	10^8^	10^9^			10^7^	10^8^	10^9^
MC1 (spores)	10^7^	9.0 × 10^−7^ *gh*	3.7 × 10^−7^ *jk*	1.7 × 10^−7^ *klmnop*	MC1 (mycelium)	10^7^	2.2 × 10^−6^ *c*	3.3 × 10^−6^ *b*	3.6 × 10^−6^ *a*
	10^8^	2.3 × 10^−7^ *klmno*	2.7 × 10^−7^ *klm*	2.1 × 10^−7^ *klmnop*		10^8^	1.9 × 10^−7^ *klmnop*	2.6 × 10^−7^ *klmn*	3.1 × 10^−7^ *jkl*
	10^9^	3.5 × 10^−8^ *nop*	2.6 × 10^−8^ *op*	1.9 × 10^−8^ *op*		10^9^	1.4 × 10^−8^ *op*	2.2 × 10^−8^ *op*	2.9 × 10^−8^ *op*

**Table 3:** Intergeneric conjugation frequencies between *E. coli* S17-1 and *Streptomyces* sp. MC1 in different conditions and with various donor/recipient ratios (CFU/plate) The mean values are given. Different letters indicate significant differences between means (*p* < 0.05).

MgCl_2_ 10 mM									
(a)	CFU/plate	*E. coli* S17-1			(b)	CFU/plate	*E. coli* S17-1		
		10^7^	10^8^	10^9^			10^7^	10^8^	10^9^
MC1 (spores)	10^7^	2.6 × 10^−5^ *de*	1.9 × 10^−5^ *f*	1.1 × 10^−5^ *h*	MC1 (mycelium)	10^7^	2.0 × 10^−5^ *f*	2.4 × 10^−5^ *e*	2.6 × 10^−5^ *d*
	10^8^	9.1 × 10^−6^ *hi*	9.3 × 10^−6^ *hi*	8.2 × 10^−6^ *i*		10^8^	1.9 × 10^−6^ *kl*	2.0 × 10^−6^ *k*	1.8 × 10^−6^ *klm*
	10^9^	1.8 × 10^−6^ *klmn*	1.5 × 10^−6^ *klmn*	1.1 × 10^−6^ *klmn*		10^9^	9.0 × 10^−8^ *n*	1.5 × 10^−7^ *mn*	1.8 × 10^−7^ *lmn*
CaCl_2_ 60 mM									
(c)	CFU/plate	*E. coli* S17-1			(d)	CFU/plate	*E. coli* S17-1		
		10^7^	10^8^	10^9^			10^7^	10^8^	10^9^
MC1 (spores)	10^7^	2.4 × 10^−5^ *e*	2.0 × 10^−5^ *f*	1.3 × 10^−5^ *g*	MC1 (mycelium)	10^7^	4.9 × 10^−5^ *c*	5.2 × 10^−5^ *b*	6 × 10^−5^ *a*
	10^8^	5.6 × 10^−6^ *j*	5.9 × 10^−6^ *j*	5.5 × 10^−6^ *j*		10^8^	4.4 × 10^−6^ *j*	4.7 × 10^−6^ *j*	4.5 × 10^−6^ *j*
	10^9^	6.5 × 10^−7^ *klmn*	6.1 × 10^−7^ *klmn*	5.3 × 10^−7^ *klmn*		10^9^	2.8 × 10^−7^ *lmn*	3.1 × 10^−7^ *lmn*	4.0 × 10^−7^ *klmn*

## Data Availability

The original contributions presented in this study are included in the article/Supplementary Material. Further inquiries can be directed to the corresponding author.

## Supplementary Materials

The following supporting information can be downloaded at: https://www.mdpi.com/article/10.3390/ijms27010093/s1.
