# Supplementary material for: Fluorescence-Based Soil Survival Analysis of the Xenobiotic- and Metal-Detoxifying Streptomyces sp. MC1
Source: Int J Mol Sci. 2025 Dec 21;27(1):93. doi: 10.3390/ijms27010093 (PMC12785999; doi:10.3390/ijms27010093)

Supplementary Materials

Fluorescence-based soil survival analysis of the xenobiotic- and metal-detoxifying *Streptomyces* sp. MC1

Juan Daniel Aparicio [1,2], Victoria Guadalupe Gonzalez Holc [1], Christhian Gabriel Pappalardo [2], Sylvie Lautru [3], Jean-Luc Pernodet [3], and Marta Alejandra Polti [1,4]

1 Planta Piloto de Procesos Industriales Microbiológicos (PROIMI), CONICET. Av. Belgrano y Pasaje Caseros. 4000. Tucumán, Argentina

2 Facultad de Bioquímica, Química y Farmacia, Universidad Nacional de Tucumán. Ayacucho 491. 4000. Tucumán, Argentina

3 Université Paris-Saclay, CEA, CNRS, Institute for Integrative Biology of the Cell (I2BC), 91198, Gif-sur-Yvette, France

4 Facultad de Ciencias Naturales e Instituto Miguel Lillo, Universidad Nacional de Tucumán. Miguel Lillo 205. 4000. Tucumán, Argentina

Figure S1. Growth of *Streptomyces* sp. MC1 in different conditions. Pictures of the plates were taken after seven days of incubation on different solid culture media: ISP4 (International *Streptomyces* Project medium 4), CSA (Casein Starch Agar), SFM (Soy Flour Mannitol), and MP5 (Medium of Production 5), at 30°C, 35°C, 40°C, 45°C and 50 °C. B: back, F: front.

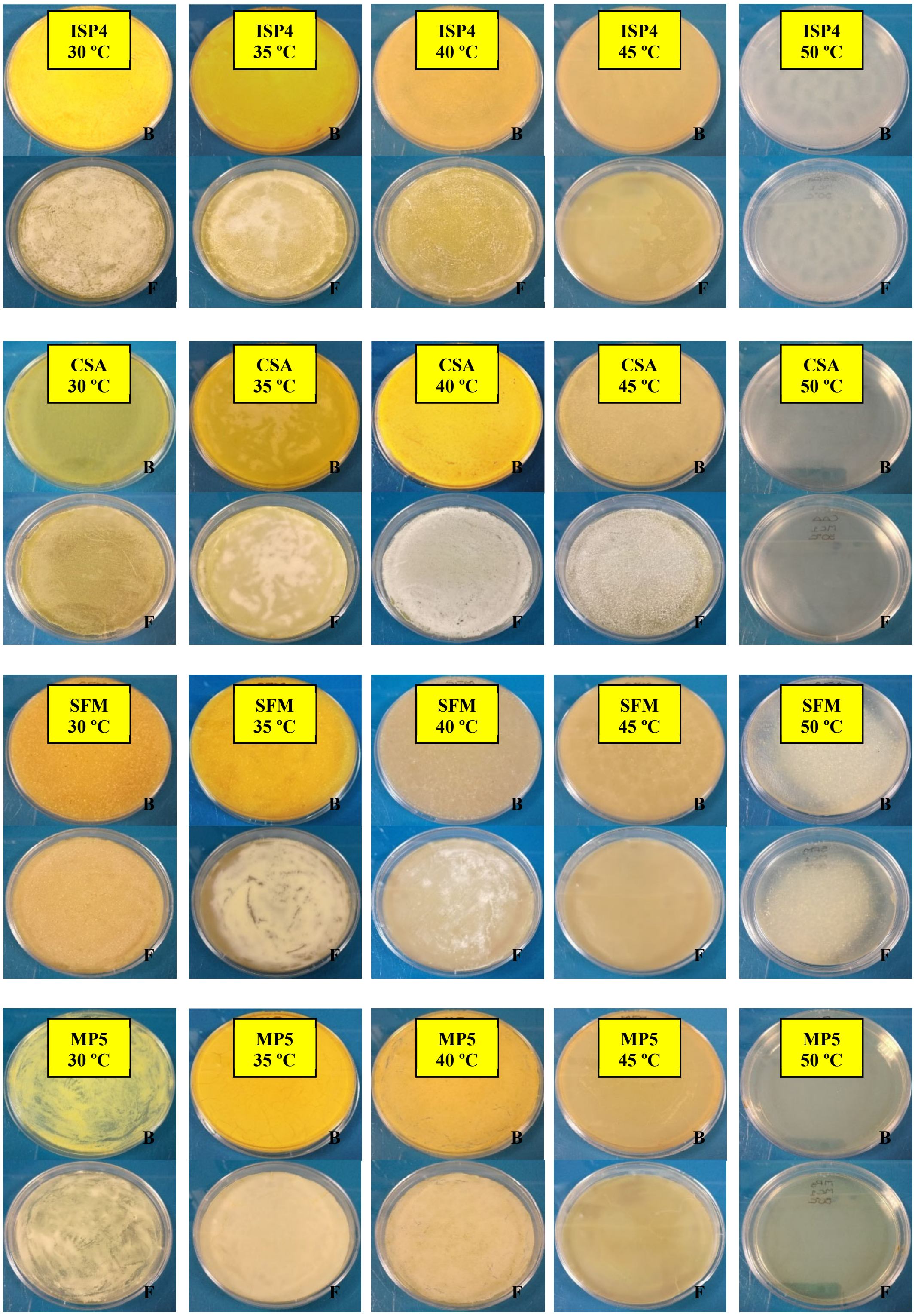

Supplement: Supplementary file 1 [file ijms-27-00093-s001.zip › ijms-4038528-supplementary.pdf]
